# Phylogenetic studies of magnoliids: Advances and perspectives

**DOI:** 10.3389/fpls.2022.1100302

**Published:** 2023-01-16

**Authors:** Zhiguo Shen, Xin Ding, Jianming Cheng, Fangfang Wu, Hengfu Yin, Minyan Wang

**Affiliations:** ^1^ National Innovation Alliance of Wintersweet, Henan Academy of Forestry, Zhengzhou, China; ^2^ Scientific Research Department, Scientific Research Department, Henan Colorful Horticulture Co., Ltd, Zhengzhou, China; ^3^ Research Institute of Subtropical Forestry, Chinese Academy of Forestry, Hangzhou, Zhejiang, China

**Keywords:** magnoliids, phylogeny, monocots, eudicots, genome

## Abstract

Magnoliids are the largest flowering plant clades outside of the eudicots and monocots, which are distributed worldwide and have high economic, ornamental and ecological values. Eudicots, monocots and magnoliids are the three major clades of Mesangiospermae, and their phylogenetic relationship is one of the most interesting issues. In recent years, with the continuous accumulation of genomic information, the evolutionary status of magnoliids has become a hot spot in plant phylogenetic research. Although great efforts have been made to study the evolution of magnoliids using molecular data from several representative species such as nuclear genome, plastid genome, mitochondrial genome, and transcriptome, the results of current studies on the phylogenetic status of magnoliids are inconsistent. Here, we systematically describe the current understanding of the molecular research on magnoliid phylogeny and review the differences in the evolutionary state of magnoliids. Understanding the research approaches and limitations of magnoliid phylogeny can guide research strategies to further improve the study of the phylogenetic evolution of magnoliids.

## Introduction

Angiosperms, also known as flowering plants, are the highest and most diverse category of the plant kingdom and have a significant dominance on Earth (Tang et al., 2014; Yang L. et al., 2020; Yang Y. et al., 2020). It has been reported that there are over 35,2000 species of angiosperms (http://www.theplantlist.org/), which are essential sources of oxygen, food, fiber, medicines and other materials for humans and animals (Judd et al., 1999; Tilman et al., 2002). Darwin referred to the phenomenon of rapid origin and species diversity of angiosperms in a relatively short geological period as an “abominable mystery” (Davies et al., 2004; Crepet and Niklas, 2009; Friedman, 2009; Buggs, 2017; Chen et al., 2017). Phylogenetic relationships among organisms are fundamental to evolutionary biology and many other disciplines (Zhang et al., 2012). The establishment of a classification system that truly reflects plant phylogeny has been an important goal of botanical and evolutionary biology research for the largest group in the plant kingdom, angiosperms (Hsu, 1984; Cronquist, 1988; Takhtajan, 1997).

Angiosperms have long been classified into two major groups: monocotyledons and dicotyledons, according to the four major classification systems of Cronquist, Takhtajan, Engler, and Hutchinson (Engler, 1964; Hutchinson, 1973; Cronquist, 1988; Takhtajan, 1997). With the development of molecular biology, the phylogenetic studies of angiosperms have made amazing progress and the taxonomic perspective of angiosperms has undergone revolutionary changes. The Angiosperm Phylogeny Group (APG), composed of several scholars, proposed the APG system based on molecular data in 1998 (Bremer et al., 1998), which is a new classification system of angiosperms based on cladistics and molecular systematics in three revised versions (Bremer et al., 2003; The APG, 2009; Chase et al., 2016). The APG system has changed the traditional phylogenetic research based on fossil records, species morphology and physiological characteristics, and has had a significant impact on the phylogenetic study of angiosperms. Today, the APG system has become a widely used classification system for angiosperms. In the most recent APG IV classification system, angiosperms are classified as basal angiosperms and Mesangiospermae (Chase et al., 2016). The basal angiosperms (ANA clade) include Amborellales, Nymphaeales and Austrobaileyales (Qiu et al., 1999), and Mesangiospermae include five branches: eudicots, monocots, magnoliids, ceratophyllales, and chloranthales. Among them, eudicots and monocots are the two most abundant groups, accounting for about 75% and 20% of angiosperm species, respectively (Zeng et al., 2014). Magnoliids are the third largest branch with more than 10,000 species, accounting for less than 3% of angiosperm species (http://www.theplantlist.org/). Chloranthales and ceratophyllales are few in number and rare in morphology, with only 77 and 6 species, respectively (Zeng et al., 2014).

Many species of magnoliids are early diverging lineages and play an important role in the study of plant evolution and phylogeny, which can be used to better understand the evolution of extant angiosperms (Massoni et al., 2015; Chen et al., 2019; Shang et al., 2020; Liu et al., 2020; Wu et al., 2021). Moreover, many species have high economic, ornamental and ecological values and are widely distributed worldwide (Massoni et al., 2014; Zeng et al., 2014; Massoni et al., 2015; Dong et al., 2021; Shen et al., 2021). Therefore, magnoliids are of great interest to botanists and plant breeders. Nevertheless, the evolutionary relationships between eudicots, monocots, and magnoliids remain unclear, and differences in topology may reveal the phylogenetic complexity behind the rapid radiation of angiosperms (Soltis and Soltis, 2019). In this paper, the phylogenetic research of magnoliids is reviewed and the potential reasons for differences in the evolutionary state of magnoliids are summarized and discussed, with a view to providing guidance for future research.

## Overview of magnoliids

The majority of phylogenetic findings support Magnoliales, Laurales, Canellales and Piperales as a branch of Mesangiospermae with early and rapid differentiation (Soltis et al., 1999; Soltis et al., 2007, Cai et al., 2006; Cantino et al., 2007; Moore et al., 2010; Qiu et al., 2010; Moore et al., 2011; Soltis et al., 2011; Ruhfel et al., 2014), but this branch differs from the Magnoliidae, as defined in the Takhtajan classification system (Takhtajan, 1997) or the Cronquist classification system (Cronquist, 1988). Giulietti et al. (2005) and Cantino et al. (2007) associated the name of Magnoliidae with this branch, while in the APG system (Bremer et al., 2003; The APG, 2009; Chase et al., 2016), with this branch being referred to as magnoliids. This paper follows the name of magnoliids in the APG system, which is equivalent to Magnoliidae in some of the literature (Cantino et al., 2007; Massoni et al., 2014; Massoni et al., 2015).

Magnoliids are the next clades of angiosperms after eudicots and monocots, including some of the “earliest angiosperms” defined in earlier studies (Zeng et al., 2014). Magnoliids have played an important role in the development of human society, with species such as black pepper (*Piper nigrum*), avocado (*Persea americana*), *Litsea cubeba* and *Chimonanthus salicifolius* having high economic value, while other species such as *C. praecox*, *Yulania denudata*, *Magnolia grandiflora*, and *Liriodendron chinense* have high ornamental value. Besides, many organisms (including various butterfly and beetle groups) are highly dependent on this group for feeding or reproduction, which is an important part of the forest ecosystem (Massoni et al., 2015). Magnoliids have morphological characteristics of both eudicots and monocots (Tang et al., 2014). For example, the *L. chinense* flower has three cardinal numbers with single pore pollen grains and exhibits typical monocot characteristics, while the cotyledons and roots show typical eudicot characteristics (Chen et al., 2019).

The phylogeny of magnoliids, monocots and eudicots is related to the early origin and evolution of angiosperms. Clarifying the phylogenetic status of magnoliids will provide a new direction for phytogenetic studies and promote the interpretation of evolutionary mysteries and the disclosure of earth history (Zhang et al., 2022). In recent years, the evolutionary status of magnoliids has become a hot spot for plant phylogenetic studies. Among the existing published studies on the phylogeny of magnoliids, most researchers have tried to explain the phylogenetic status of magnoliids using nuclear genomes and other molecular data (plastid genomes, mitochondrial genomes, transcriptomes, etc.) of several representative species, but the conclusions are inconsistent. In general, the topological structure of the evolutionary relationships between the three clades include the following three types: (1) magnoliids + (eudicot + monocot); (2) monocot + (eudicot + magnoliids); (3) eudicot + (magnoliids + monocot).

## Phylogeny based on the sequencing of magnoliids own nuclear genome

Plant cells contain three sets of genomes: the nuclear genome, the plastid genome and the mitochondrial genome. The nuclear whole-genome sequences contain rich genetic information and have greater application potential in phylogenetic research, with potent capabilities to decipher complex phylogenetic models and evolutionary processes, which can deepen our understanding of plant phylogeny and evolution. The reported species of magnoliids with sequenced nuclear genomes include Magnoliales, Laurales, and Piperales (17 species, 23 references in total). Examples include Laurales: *Cinnamomum kanehirae* (Chaw et al., 2019), avocado (*Persea americana*) (Rendón-Anaya et al., 2019; Nath et al., 2022), *Litsea cubeba* (Chen Y. C. et al., 2020), *Phoebe bournei* (Chen S. P. et al., 2020; Han et al., 2022), *Cinnamomum camphora* (Jiang et al., 2022; Shen et al., 2022; Sun et al., 2022; Wang et al., 2022), *C. burmannii* (Li et al., 2022), *Litsea coreana* (Zhang et al., 2022) of Lauraceae, *Chimonanthus praecox* (Shang et al., 2020; Shen et al., 2021) and *C. salicifolius* (Lv et al., 2020) of Calycanthaceae; Magnoliales: *L. chinense* (Chen et al., 2019), *Magnolia biondii* (Dong et al., 2021), *M. officinalis* (Yin et al., 2021); *Annona muricata* (Strijk et al., 2021), *A. glabra* (He et al., 2022); Pepperales: *Piper nigrum* (Hu et al., 2019), *Aristolochia fimbriata* (Qin et al., 2021) and *A. contorta* (Cui et al., 2022). Twenty of the 23 references in the genome analysis magnoliids discussed the evolutionary status of magnoliids, providing new insights into the early evolution of angiosperms, but the results of the analysis are inconsistent.

### Magnoliids form a sister clade to monocots and eudicots

The diversity of rapid formation or differentiation of common ancestors of magnoliids, monocots and eudicots leads to differences in the topological results of phylogenetic trees (incomplete lineage sorting, ILS). Chen et al. (2019) sequenced the nuclear genome of *L. chinense* and deduced three topological structures based on 502 low-copy (no more than two per species) gene trees from 17 species. The species tree was further constructed by the amino acid coalescence approach, and further topological analysis of 78 chloroplast genes and gene families specific to monocots and eudicots in the Liriodendron genome was performed, respectively, which all supported that magnoliids are sister plants to monocots and eudicots. Hu et al. (2019), based on nuclear genome sequencing of *P. nigrum* and 82 single-copy genes identified in 21 species, used the amino acid concatenation approach to support that magnoliids form a sister clade to monocots and eudicots. Rendón-Anaya et al. (2019) sequenced the nuclear genome of avocado and determined 176 single-copy genes from 19 species. Based on protein sequences, avocado is considered to be sister to monocots and eudicots; based on CDS sequences, avocado is sister to monocots; also, based on 4,694 low-copy genes, avocado is considered to be sister to eudicots. Besides, a neighbor-joining tree was generated based on modal dissimilarity scores from thousands of syntenically validated ortholog pairs, indicating that avocado is sister plants to monocots and eudicots. By evaluating the three positions of avocado, the authors concluded that the three different positions of avocado in angiosperms may be indistinguishable for purely biological reasons. However, according to the Akaike information criterion (AIC) comparison based on the free rate (FR) model, avocado is preferred as sister plants to monocots and eudicots. Chen S. P. et al. (2020) sequenced the nuclear genome of *P. bournei* and constructed five evolutionary trees based on 292 single-copy genes from 18 species. Among them, three trees (Bayesian tree, coalescent and concatenation trees based on amino acid sequences) support that magnoliids form a sister clade to monocots and eudicots; two trees (coalescent and concatenation trees based on nucleotide sequences) support that magnoliids are sister to monocots. The authors support the Bayesian tree. Zhang et al. (2022) sequenced the nuclear genome of *L. coreana*. Using 71 single-copy genes of the nuclear genome from 13 species (*Amborella trichopoda* as an outgroup), nucleic acid sequence-based and amino acid sequence-based concatenation trees were constructed to support that magnoliids are sister to eudicots; the constructed amino acid sequence-based coalescent tree supports that magnoliids form a sister clade to monocots and eudicots and that black pepper is closer to monocots and eudicots. The authors concluded that *Magnoliids* are more likely to be the basal species of angiosperms due to the possibility of ILS. Consistently, the results of nuclear genome sequencing analysis of *M. officinalis*, *M. biondii*, *C. camphora*, etc. support that magnoliids form a sister clade to monocots and eudicots (Dong et al., 2021; Yin et al., 2021; Jiang et al., 2022).

### Magnoliids are sister to eudicots

Chaw et al. (2019) sequenced the nuclear genome of *C. kanehirae*. Using 211 single-copy genes determined from 13 species, amino acid sequence-based coalescent and concatenation trees support the idea that magnoliids are sister to eudicots. Meanwhile, this topology is also supported by transcriptome data from 22 magnoliids species (although the BS is somewhat low). Similarly, in the genome sequencing analysis of *C. salicifolius*, Lv et al. (2020) constructed an amino acid sequence-based concatenation tree of 103 single-copy gene sets and coalescent tree of 1,420 low-copy gene sets from 17 species, all of which support that magnoliids are sister plants to eudicots. In addition, Shang et al. (2020) used two software (OrthoMCL and SonicParanoid) to identify two single-copy gene sets and construct amino acid sequence-based concatenation and coalescent trees, respectively. A total of four trees support that magnoliids are sister plants to eudicots. However, based on 38 chloroplast single-copy genes from 26 species, the results of amino acid sequence-based concatenation tree support that magnoliids form a sister clade to monocots and eudicots. The authors suggest that the phylogenetic inconsistency between chloroplast genomes and nuclear genomes may be caused by the ILS effect. Furthermore, a concatenation phylogenetic tree was constructed using nucleic acid sequences of 2,420 gene sets from 29 plants (including transcriptome data), again demonstrating that magnoliids are sister plants to eudicots. Therefore, the authors believe that it is relatively accurate that magnoliids are the sister plants to eudicots in the current data set. In the nuclear genomic analysis of the red flower wintersweet, taking into full consideration various factors that may affect the evolutionary position of magnoliids, Shen et al. (2021) constructed concatenation and coalescent trees using nucleic acid and amino acid sequences of 70 single-copy gene families from 25 genomes, as well as phylogeny trees of 123 plants (47 transcripts, 76 genomes) based on the nucleotide sequences of selected low-copy nuclear ortholog groups. The results suggest that magnoliids are more likely to form a sister clade to eudicots, which is supported by more phylogenetic trees. Consistently, the results of nuclear genome sequencing analysis of *C. camphora*, *A. glabra, P. bournei, A. contorta*, *C. burmannii*, etc. support that magnoliids are sister plants to eudicots (Cui et al., 2022; Han et al., 2022; He et al., 2022; Li et al., 2022; Shen et al., 2022; Sun et al., 2022; Wang et al., 2022).

### Magnoliids are sister to monocots or the evolutionary relationships remain unresolved

Qin et al. (2021) compared the genome structure of *A. fimbriata* and representative species of major angiosperm groups and placed magnoliids as sister groups of monocots. Chen Y. C. et al. (2020) sequenced the *L. cubeba* nuclear genome, obtained 160 common single-copy gene families of 34 angiosperms from the BUSCO database, and constructed concatenation and coalescent trees using nucleic acid and amino acid sequences. Among them, the amino acid sequence-based coalescent tree supports that magnoliids are sister plants to monocots, and the other three trees support that magnoliids are sister plants to eudicots. Analysis by ASTRAL software suggested that a possible ILS effect on the rapid differentiation of early Mesangiospermae. Based on this, the authors conclude that the evolutionary relationships between magnoliids, monocots, and eudicots remain unresolved.

To sum up, among the 23 references for phylogenetic analysis based on sequencing of magnoliids own nuclear genome, 8 references supported magnoliids as a sister clade to monocots and eudicots, 11 references supported magnoliids as a sister clade to eudicots, and 1 reference supported magnoliids as a sister clade to monocots. In addition, the authors of one reference considered that the evolutionary relationships between magnoliids, monocots and eudicots remain unresolved (**Table 1**).

**Table 1:** Phylogeny based on the sequencing of magnoliids own nuclear genome.

Phylogeny of magnoliids	Author	Species	Order	Family	Journal	Year of publication
Magnoliids form a sister clade to monocots and eudicots (8 references)	Rendón-Anaya et al.	*Persea americana*	Laurales	Lauraceae	*PNAS*	2019
	Chen et al.	*Phoebe bournei*	Laurales	Lauraceae	*Hortic. Res.*	2020
	Yin et al.	*Magnolia officinalis*	Magnoliales	Magnoliaceae	*iScience*	2021
	Dong et al.	*Magnolia biondii*	Magnoliales	Magnoliaceae	*Hortic. Res.*	2021
	Chen et al.	*Liriodendron chinense*	Magnoliales	Magnoliaceae	*Nat. Plants*	2019
	Hu et al.	*Piper nigrum*	Piperales	Piperaceae	*Nat. Commun.*	2019
	Jiang et al	*Cinnamomum camphora*	Laurales	Lauraceae	*Front. plant sci.*	2022
	Zhang et al	*Litsea coreana*	Laurales	Lauraceae	*Genomics*	2022
Magnoliids are sister plants to eudicots (11 references)	Chaw et al.	*Cinnamomum kanehira*	Laurales	Lauraceae	*Nat. Plants*	2019
	Sun et al.	*Cinnamomum camphora*	Laurales	Lauraceae	*J. Genet. Genomics*	2022
	Li et al.	*Cinnamomum burmannii*	Laurales	Lauraceae	*Ind. Crop. Prod.*	2022
	Lv et al.	*C. salicifolius*	Laurales	Calycanthaceae	*Plant J.*	2020
	Shang et al.	*Chimonanthus praecox*	Laurales	Calycanthaceae	*Genome Biol.*	2020
	Shen et al.	*C. praecox* ‘Hongyun’	Laurales	Calycanthaceae	*Plant J*	2021
	Cui et al.	*Aristolochia contorta*	Piperales	Aristolochiaceae	*Hortic. Res.*	2022
	He et al	*Annona glabra*	Magnoliales	Annonaceae	*Nat. Eco. & Evol.*	2022
	Han et al.,	*Phoebe bournei*	Laurales	Lauraceae	*Plant Commun.*	2022
	Wang et al	*Cinnamomum camphora*	Laurales	Lauraceae	*Hortic. Res.*	2022
	Shen et al	*Cinnamomum camphora*	Laurales	Lauraceae	*Plant Biotechnol. J.*	2022
Magnoliids are sister plants to monocots (1 reference)	Qin et al.	*Aristolochia fimbriata*	Piperales	Aristolochiaceae	*Nat. Plants*	2021
The evolutionary relationships remain unresolved (1 reference)	Chen et al.	*Litsea cubeba*	Laurales	Lauraceae	*Nat. Commun.*	2020
No phylogenetic analysis of magnoliids (2 references)	Strijk et al.	*Annona muricata*	Magnoliales	Annonaceae	*Mol. Ecol. Resour.*	2021
	Nath et al.	*Persea americana*	Laurales	Lauraceae	*Hortic. Res.*	2022

## Phylogeny of magnoliids based on other molecular data

Over the years, researchers have also integrated plastid, mitochondrial, nuclear genome and transcriptome molecular data from multiple species to analyze the phylogeny of magnoliids and the early diversification of angiosperms. Different phylogenetic relationships have also emerged regarding the status of magnoliids.

### Magnoliids form a sister clade to monocots and eudicots

In terms of phylogenetic analysis using plastid genomes, Cai et al. (2006); Moore et al. (2007), and Ruhfel et al. (2014) conducted phylogenetic analysis of 61 plastid protein-coding genes from 35 taxa, 61 plastid protein-coding genes from 45 species, and 78 plastid protein-coding data from 360 species, respectively; Moore et al. (2010) conducted a phylogenetic analysis of 83 protein-coding and rRNA genes from 86 seed plant plastid genomes. Gitzendanner et al. (2018) analyzed the phylogenetic tree of 1,827 green plants and 52 outgroups using 78 plastid protein-coding genes. Li et al. (2019) reconstructed the angiosperm phylogeny based on 80 genes from 2,881 plastid genomes, representing 85% of extant families and all orders. In terms of phylogenetic analysis using the mitochondrial genome, Qiu et al. (2010) performed a phylogenetic analysis of 380 species of seed plants based on four mitochondrial gene sequences. Dong et al. (2020) conducted a phylogenetic analysis based on 38 mitochondrial genes from 91 representative angiosperm species. In addition, Soltis et al. (2011) conducted a two-group analysis of 640 plant species from 330 families. The first group included 17 genes representing all three plant genomes (i.e., nucleus, plastid, and mitochondrion); the second group contained 13 genes (representing only the nucleus and plastid). Jin et al. (2020) constructed 20 phylogenetic trees based on nucleotide and amino acid sequences of five gene sets from 89 plants using concatenation and coalescent approaches. The results of all the above analyses support that magnoliids form a sister clade to monocots and eudicots.

### Magnoliids are sister to eudicots

Moore et al. (2011) analyzed the plastid inversion repeat sequences of 244 plants; Zeng et al. (2014) and Puttick et al. (2018) conducted phylogenetic analysis using transcriptome amino acid sequences from 61 and 103 plants, respectively; Wickett et al. (2014) systematically analyzed 852 protein-coding nuclear genes from 103 plants (92 transcriptomes and 11 nuclear genomes). The results of the above analysis support that magnoliids are sister plants to eudicots. In addition, Zhang et al. (2020) constructed a coalescent tree based on five different low-copy gene sets (comprising 1,167, 834, 683, 602, and 445 genes respectively) from 115 plants (44 nuclear genomes and 71 transcriptomes), most of which support that magnoliids are sister plants to eudicots. Guo et al. (2021) constructed phylogenetic trees based on nuclear genome sequencing of *Chloranthus spicatus* using four gene sets (257 single-copy genes, 937 single-copy genes, and 2,329 low-copy genes from 18 plants, and 612 single-copy genes from 218 plants, respectively), supporting the idea that magnoliids are sister to eudicots, while the results of chloroplast gene construction support that magnoliids form a sister clade to monocots and eudicots. The analysis suggested that ancient gene flow between monocots and eudicots might have occurred during the early evolution of angiosperms, resulting in inconsistent phylogenetic branches. In addition, Ma et al. (2021) sequenced the nuclear genome of *C. sessilifolius* and analyzed 1,689 single-copy genes concatenated nucleotide sequences based on nuclear genome data from 14 plants, supporting that magnoliids are sister plants to eudicots. At the same time, the analysis suggests that, in addition to hybridization, ILS may largely explain the observed phylogenetic inconsistencies among gene trees.

### Magnoliids are sister to monocots or the evolutionary relationships remain unresolved

Zhao et al. (2021) conducted a phylogenetic analysis based on genome-wide data from 123 plants (covering 31 orders and 52 families); Zhang et al. (2012) constructed a concatenation tree using nucleotide and amino acids based on five low-copy nuclear genes obtained in 94 species; Endress and Doyle (2009) analyzed plastid and morphological data. The results of all these analyses suggest that magnoliids are sister plants to monocots. However, based on 1594, 756, and 296 gene sets from 151 angiosperms (including the five major branches of the core angiosperms), Yang L. et al. (2020) employed both coalescent and concatenation approaches to infer phylogenetic trees of angiosperms. The authors believe that a fully bifurcated species tree may not be the best way to represent the early differentiation of angiosperms.

To sum up, among the 21 references on the phylogenetic analysis of magnoliids, 10 support that magnoliids form a sister clade to monocots and eudicots, 7 support that magnoliids form a sister clade to eudicots, 3 support that magnoliids form a sister clade to monocots, and one believes that the evolutionary relationships between magnoliids, monocots, and eudicots remain unresolved (**Table 2**).

**Table 2:** Phylogeny of magnoliids based on other molecular data.

Phylogeny of magnoliids	Author	Journal	Year of publication
Magnoliids form a sister clade to monocots and eudicots (10 references)	Cai et al.	*BMC Evol. Biol.*	2006
	Moore et al.	*PNAS*	2007
	Moore et al.	*PNAS*	2010
	Soltis et al.	*Am. J. Bot.*	2011
	Qiu et al.	*J. Syst. & Evol.*	2010
	Ruhfel et al.	*BMC Evol. Biol.*	2014
	Li et al.	*Nat. Plants*	2019
	Gitzendanner et al.	*Am. J. Bot.*	2018
	Dong et al.	*PloS one*	2020
	Jin et al.	*Guihaia*	2020
Magnoliids are sister plants to eudicots (7 references)	Moore et al.	*Inter. J. Plant Sci.*	2011
	Zeng et al.	*Nat. Commun.*	2014
	Wickett et al.	*PNAS*	2014
	Puttick et al.	*Curr. Biol.*	2018
	Zhang et al.	*Nature*	2020
	Sun et al.	*J. Genet. genomics*	2022
	Ma et al.	*Nat. Commun.*	2021
Magnoliids are sister plants to monocots (3 references)	Zhao et al.	*Nat. Commun.*	2021
	Zhang et al.	*New Phytol.*	2012
	Endress et al.	*Am. J. Bot.*	2009
The evolutionary relationships remain unresolved (1 reference)	Yang et al.	*Plant Commun.*	2020

## Summary and perspectives

Magnoliids have important economic, ornamental and ecological values (Massoni et al., 2014; Massoni et al., 2015; Shen et al., 2021; Dong et al., 2021). They are also valuable materials for studying the origin, development and evolution of angiosperms (Massoni et al., 2015; Chen et al., 2019; Liu et al., 2020; Shang et al., 2020; Wu et al., 2021). Despite the large number of studies reporting the phylogenetic status of magnoliids, the evolutionary relationships between eudicots, monocots, and magnoliids remain inconsistent (**Tables 1**, **2**). Long-branch attraction is a major obstacle to phylogenetic reconstruction, which may lead to the wrong inference of distantly related lineages as close relatives (Qu et al., 2017; Shen et al., 2021). Meanwhile, ILS is the result of allele polymorphism in ancestral populations (Chen Y. C. et al., 2020). Many plant species have a century-long growth period, large population sizes, and limited interspecific differences. These factors have generated an important evolutionary network, which is deeply affected by the ILS. In addition, more attention should be paid to methodological choices in phylogenomic analysis, where the same data set may yield conflicting results (Guo et al., 2022). Different tree-building methods may be important factors contributing to the different evolutionary positions of magnoliids (Rendón-Anaya et al., 2019; Chen S.P. et al., 2020; Chen Y. C. et al., 2020; Shen et al., 2021). It is precisely because of the different tree-building methods, the existence of ILS effects, the number of orthologous genes, the limitation of numerical selection in different groups (Bergsten, 2005; Wiens, 2005), and the rapid differentiation of magnoliids in the early evolutionary stage that the results of research on the evolutionary status of magnoliids are different.

A fully resolved and well-supported phylogeny is of great significance for understanding the evolutionary history of magnoliids. Based on the comprehensive analysis of existing research results, how to adopt a more scientific strategy to analyze the phylogeny of magnoliids is a key consideration for future research on the evolution of magnoliids. For a long time, a large number of valuable plant species have not been sequenced due to the cost of sequencing and the complexity of the species’ own genomes. In particular, there are still few genome sequencing samples of magnoliids, which also hinders the in-depth study of these issues to a certain extent. With the rapid development of sequencing technology and the reduction of sequencing cost, an increasing number of plant genome sequencing data will be published, especially more genomic data of magnoliids will be deciphered, and with the more mature means of phylogenetic research, it is believed that in the near future, there will be an industry-recognized result on the phylogenetic status of magnoliids.

## Author contributions

ZS was the designer and principal of the project, and drafted the manuscript. XD, JC, and FW were responsible for collecting, sorting, and analyzing some relevant references. HY and MW contributed to the manuscript revision and read the submitted version. All authors contributed to the article and approved the submitted version.
